# Resection of Early-Stage Second Primary Non-small Cell Lung Cancer After Small Cell Lung Cancer: A Population-Based Study

**DOI:** 10.3389/fonc.2019.01552

**Published:** 2020-02-05

**Authors:** Rusi Zhang, Ling Cai, Gongming Wang, Yingsheng Wen, Fang Wang, Ningning Zhou, Xuewen Zhang, Zirui Huang, Xiangyang Yu, Kexing Xi, Longjun Yang, Dechang Zhao, Yongbin Lin, Lanjun Zhang

**Affiliations:** ^1^State Key Laboratory of Oncology in South China, Collaborative Innovation Center for Cancer Medicine, Guangzhou, China; ^2^Department of Thoracic Surgery, Sun Yat-sen University Cancer Center, Guangzhou, China; ^3^Zhongshan School of Medicine, Sun Yat-sen University, Guangzhou, China; ^4^Department of Radiation Oncology, Sun Yat-sen University Cancer Center, Guangzhou, China; ^5^Department of Molecular Pathology, Sun Yat-sen University Cancer Center, Guangzhou, China; ^6^Department of Medical Oncology, Sun Yat-sen University Cancer Center, Guangzhou, China; ^7^Department of Anesthesiology, Sun Yat-sen University Cancer Center, Guangzhou, China; ^8^Department of Thoracic Surgical Oncology, National Cancer Center/National Clinical Research Center for Cancer/Cancer Hospital, Chinese Academy of Medical Sciences and Peking Union Medical College, Beijing, China; ^9^Department of Colorectal Surgery, National Cancer Center/National Clinical Research Center for Cancer/Cancer Hospital, Chinese Academy of Medical Sciences and Peking Union Medical College, Beijing, China

**Keywords:** small cell lung cancer, non-small cell lung cancer, second primary lung cancer, surgery, survival, SEER database

## Abstract

**Introduction:** A certain number of small cell lung cancer (SCLC) patients become long-term survivors after treatment, and they are at high risk to develop a second primary malignancy, including non-small cell lung cancer. However, the optimal management of early-stage second primary non-small cell lung cancer (SPLC) after SCLC remains unknown. This study aims to evaluate the survival benefits of surgery in these patients.

**Methods:** Patients with early-stage SPLC after SCLC were identified from the Surveillance, Epidemiology, and End Results database. Patients were balanced with propensity score matching (PSM). Overall survival (OS) and lung cancer-specific survival (CSS) were compared between non-surgery group and surgery group with the Kaplan–Meier method and Cox multivariate regressions.

**Results:** A total of 228 patients with early-stage SPLC after SCLC were identified. Surgery was associated with significantly improved OS and CSS in the multivariate Cox regression analysis (OS, 5-year survival: 41.2 vs. 11.6%, HR: 0.42, 95% CI: 0.31–0.59, *P* < 0.01; CSS, 5-year survival: 46.8 vs. 24.3%, HR: 0.53, 95% CI: 0.37–0.75, *P* < 0.01). However, no statistically significant survival difference was found between sublobar resection and lobectomy (OS, 5-year survival: 41.0 vs. 45.3%, *P* = 0.73; CSS, 5-year survival: 43.5 vs. 54.1%, *P* = 0.49). After 1:1 PSM, 162 patients were selected for further analysis, and surgery continued to demonstrate superior survival (OS, 5-year survival: 44.2 vs. 7.2%, HR: 0.48, 95% CI: 0.33–0.70, *P* < 0.01; CSS, 5-year survival: 48.0 vs. 20.6%, HR: 0.44, 95% CI: 0.42–0.97, *P* = 0.03).

**Conclusion:** The resection of early-stage SPLC after SCLC led to significantly improved OS and CSS and therefore should be considered whenever possible. Nevertheless, further randomized controlled trials are warranted to investigate the safety and effect of surgery in these patients.

## Introduction

Small cell lung cancer (SCLC) is an important type of lung cancer and accounts for ~13% of all lung cancer (1). SCLC is notorious for its aggressive behavior, and most SCLC is initially diagnosed as extensive-stage disease. Chemotherapy and radiotherapy are currently the predominant treatment modalities for SCLC due to its chemoradiosensitivity. The prognosis of SCLC patients is bleak with a 5-year survival rate of only 6% (2, 3). However, a certain number of SCLC patients become long-term survivors after radiotherapy and chemotherapy. Previous studies have demonstrated that these patients are at high risk for developing a second primary malignancy, including non-small cell lung cancer (NSCLC), possibly related to the secondary effect of chemoradiation (4, 5). In addition, It is well-established that different histology is one of the most robust criteria for the diagnosis of second primary lung cancer, which are endorsed by both International Association for the Study of Lung Cancer (IASLC) TNM staging proposal and American College of Chest Physicians (ACCP) guideline (6, 7). Therefore, it is reasonable to consider NSCLC diagnosed after SCLC as second primary lung cancer because these two cancers are different in histology.

Currently, no clinical guideline or consensus has addressed the optimal treatment strategy for early-stage second primary non-small cell lung cancer (SPLC) after SCLC. In addition, to the best of our knowledge, no previous study has evaluated the survival benefits of surgical resection for these patients.

In this study, we searched the Surveillance, Epidemiology, and End Results (SEER) database to analyze the characteristics of early-stage SPLC after SCLC, and we aimed to evaluate the survival benefits of surgery for these patients.

## Materials and Methods

### Study Population and Outcomes

Patients with early-stage SPLC after SCLC were identified from the SEER Multiple Primary Standardized Incidence Ratios (MP-SIR) session (April 2019 released, 2000–2016). A minimal interval of 6 months was required between initial SCLC and SPLC, considering the general treatment and surveillance protocol of SCLC.

Patients' clinicopathological characteristics, treatment information, and survival outcomes were also extracted from SEER. Tumor histology was classified with the International Classification of Diseases for Oncology, 3rd Edition (ICD-O-3) morphology codes according to the 2015 World Health Organization Classification of Lung Tumors (8). Specific inclusion criteria were listed as follows: (1) ICD-O-3 topographical codes: C340–C349 (lung and bronchus); (2) ICD-0-3 histology codes of the first primary lung cancer: 8002, 8041–8045 (small cell lung cancer); (3) age at diagnosis of SCLC: no less than 18 years old; (4) time of diagnosis of SPLC: 2000.1–2014.12. On the other hand, specific exclusion criteria were listed as follows: (1) ICD-O-3 histology site of the second primary lung cancer: 8002, 8041–8045 (small cell lung cancer as SPLC was excluded); (2) interval between the two cancers was less than 6 months; (3) cause of death or TNM staging information unknown; (4) N2/3 station lymph node involvement or distant metastasis. Surgery on SPLC included sublobar resection (wedge resection, segmentectomy, or other unspecified resections of less than one lobe), lobectomy, bilobectomy, pneumonectomy, and other non-specified local resection. Therefore, all selected SPLC patients with SEER Site-Specific Surgery of Primary Site Code between 10 and 90 were classified as surgery group, while Code 00 and 99 were classified as non-surgery group.

The primary and secondary outcomes in this study were overall survival (OS) and lung cancer-specific survival (CSS), respectively. The survival time was calculated from the time of SPLC diagnosis to the time of death or the last follow-up in months. All patients were followed up to December 31, 2016; patients who were alive at the last follow-up were censored. In addition, causes of death other than lung cancer were also censored in the CSS analysis.

### Statistical Analysis

The differences between the non-surgery group and the surgery group were compared by Pearson chi-square tests or Fisher's exact tests. Multiple comparisons were adjusted by the Bonferroni correction. The Kaplan–Meier method and log-rank tests were applied in survival analysis. Potential statistically significant factors (*P* < 0.10) identified from the univariate survival analysis were further selected into the Cox proportional hazards regression model for the multivariate survival analysis and subgroup analysis. The Cox regression model was developed by forward stepwise selection (likelihood-ratio) with entry and removal probabilities of 0.05 and 0.10, respectively. For the subgroup analysis, variables deemed clinically significant or those that demonstrated a survival difference in the previous multivariate Cox regression were included. Surgery for SPLC served as the comparing factor while the other included variables served as subgroup variables in turn.

Propensity score matching (PSM) was performed between the non-surgery group and the surgery group to reduce potential bias in this retrospective study. Variables deemed clinically significant and variables significantly different between the non-surgery group and the surgery group were selected as the predictor covariates in PSM. The match tolerance (caliper) was set at 0.02 and 1:1 nearest neighbor matching was performed. In this study, PSM sampled without replacement and implemented logistic regression (9).

All statistical analysis and PSM were conducted with IBM SPSS Statistics Version 25, and the survival curves and forest plot were drawn by R version 3.6.1. A two-sided *P-*value < 0.05 was considered statistically significant.

## Results

**Figure 1:**
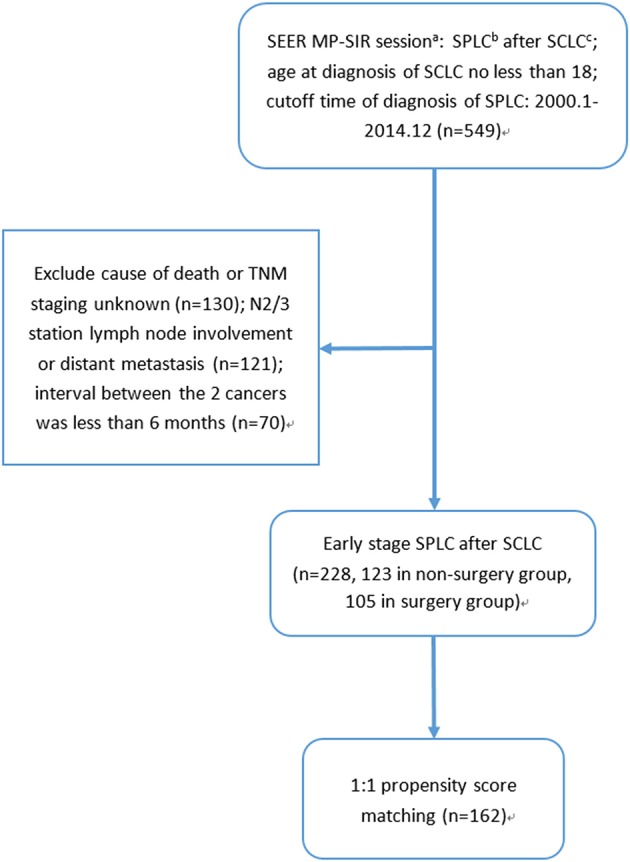
Selection flow: (a) SEER MP-SIR: Surveillance, Epidemiology, and End Results (SEER) Multiple Primary Standardized Incidence Ratios (MP-SIR) session specializes in conducting an analysis examining multiple subsequent cancers. (b) SPLC: second primary non-small cell lung cancer after small cell lung cancer. (c) SCLC: small cell lung cancer.

A total of 228 early-stage SPLC after SCLC patients were identified according to the selection criteria, among which 105 received surgery while 123 did not (selection flow, Figure 1). For the surgery group, the interval between the two cancers and the median follow-up time were 37 and 36 months, respectively; as for the non-surgery group, the interval between the two cancers and the median follow-up time were 47 and 21 months, respectively.

The pertinent clinicopathological factors were compared between the non-surgery group and the surgery group, and patients who underwent surgery were more likely to be younger and married. The surgery group was associated with limited stage SCLC, SPLC of smaller tumor size, and shorter interval between cancers. On the other hand, the histology and differentiation grade in the non-surgery group were more likely to be unclassified. Moreover, patients in the non-surgery group were more likely to receive chemotherapy and radiotherapy for SPLC. However, proportions of previous chemotherapy and radiotherapy for the initial SCLC were not significantly different between the two groups (Table 1).

**Table 1:** Comparison of clinicopathological factors between the non-surgery group and surgery group.

		**Non-surgery group**		**Surgery group**		***P***
		***n* = 123**	**Percentage**	***n* = 105**	**Percentage**	
Age	≤70 years old	63	51.2%	71	67.6%	0.01
	>70 years old	60	48.8%	34	32.4%	
Gender	Female	71	57.7%	58	55.2%	0.71
	Male	52	42.3%	47	44.8%	
Ethnicity^@^^,^^*^	Caucasian	108	87.8%	96	91.4%	0.54
	African American	12	9.8%	6	5.7%	
	Others	3	2.4%	3	2.9%	
Marital status at SPLC^a^	Married	52	42.3%	64	61.0%	0.01
	Not married	71	57.7%	41	39.0%	
Interval between SPLC and SCLC^b^	≤48 months	65	52.8%	69	65.7%	0.05
	>48 months	58	47.2%	36	34.3%	
SPLC laterality	Left	54	43.9%	45	42.9%	0.87
	Right	69	56.1%	60	57.1%	
Laterality relationship between SPLC and SCLC^@^^,^^*^	Same	57	46.3%	47	44.8%	0.97
	Different	62	50.4%	55	52.4%	
	NA	4	3.3%	3	2.9%	
SPLC primary site^@^^,^^*^	Upper lobe	86	69.9%	61	58.1%	0.24
	Middle lobe	3	2.4%	6	5.7%	
	Lower lobe	32	26.0%	35	33.3%	
	Main bronchus/Overlapping lesion/Unknown	2	1.6%	3	2.9%	
SCLC primary site^@^^,^^*^	Upper lobe	72	58.5%	61	58.1%	0.83
	Middle lobe	3	2.4%	3	2.9%	
	Lower lobe	21	17.1%	22	21.0%	
	Main bronchus/Overlapping lesion/Unknown	27	22.0%	19	18.1%	
SPLC tumor size	≤2 cm	57	46.3%	65	61.9%	0.02
	>2 cm	66	53.7%	40	38.1%	
Lymph node involvement	No	114	92.7%	99	94.3%	0.63
	Yes	9	7.3%	6	5.7%	
SPLC stage	IA–IB	95	77.2%	91	86.7%	0.07
	IIA–IIIA	28	22.8%	14	13.3%	
SPLC histology	Adenocarcinoma	36	29.3%	39	37.1%	0.04
	Squamous cell carcinoma	54	43.9%	52	49.5%	
	Others	33	26.8%	14	13.3%	
SPLC differentiation grade^*^	Well/moderately differentiated	23	18.7%	58	55.2%	<0.01
	Poorly differentiated/undifferentiated	33	26.8%	37	35.2%	
	unknown	67	54.5%	10	9.5%	
SPLC chemotherapy	No	88	71.5%	91	86.7%	<0.01
	Yes	35	28.5%	14	13.3%	
SPLC radiotherapy	No	41	33.3%	98	93.3%	<0.01
	Yes	82	66.7%	7	6.7%	
SCLC stage	Limited stage	92	74.8%	94	89.5%	<0.01
	Extensive stage	31	25.2%	11	10.5%	
SCLC surgery	No	106	86.2%	88	83.8%	0.62
	Yes	17	13.8%	17	16.2%	
SCLC chemotherapy	No	15	12.2%	13	12.4%	0.97
	Yes	108	87.8%	92	87.6%	
SCLC radiotherapy	No	34	27.6%	25	23.8%	0.51
	Yes	89	72.4%	80	76.2%	

a SPLC, second primary non-small cell lung cancer after small cell lung cancer;

b SCLC, small cell lung cancer;

@ At least one of the cells had expected cell count <5, Fisher's exact test was used;

* *Multiple comparisons were adjusted by Bonferroni correction*.

In the univariate survival analysis, patients with SPLC ≤ 2 cm, without N1 lymph node involvement, and who underwent surgery for SPLC were associated with both better OS and CSS. Surgery for SCLC demonstrated survival benefit in OS but not in CSS. Meanwhile, unclassified SPLC histology and differentiation grades were associated with worse OS and CSS. As for multivariate Cox regression, surgery for SPLC was associated with significantly improved survival (OS, 5-year rate: 41.2 vs. 11.6%, HR: 0.42, 95% CI: 0.31–0.59, *P* < 0.01; CSS, 5-year rate: 46.8 vs. 24.3%, HR: 0.53, 95% CI: 0.37–0.75, *P* < 0.01). In addition, a longer interval between cancers, SPLC ≤ 2 cm, SPLC without N1 lymph node involvement, and surgery for SCLC were also associated with significantly longer survival (Table 2).

**Table 2:** Univariate and multivariate Cox regression of early-stage SPLC after SCLC.

**Variables^a^**		**Univariate analysis**					**Multivariate analysis**			
		**OS**			**CSS**		**OS**		**CSS**	
		***n***	**HR (95% CI)**	***P***	**HR (95% CI)**	***P***	**HR (95% CI)**	***P***	**HR (95% CI)**	***P***
Age	≤70 years old	134		0.97		0.25		0.87		0.31
	>70 years old	94								
Gender	Female	129		0.13		0.10		0.23		0.11
	Male	99								
Ethnicity	Caucasian	204		0.75		0.79				
	African American	18		0.48		0.50				
	Others	6		0.77		0.90				
Interval between cancers	≤48 months	134		0.36		0.07	Reference	0.04	Reference	<0.01
	>48 months	94					0.71 (0.52–0.99)		0.61 (0.42–0.88)	
Marital status	Married	116		0.93		0.8				
	Not married	112								
Laterality	Left	99		0.12		0.41				
	Right	129								
Primary site	Upper lobe	147		0.73		0.91				
	Middle lobe	9		0.99		0.65				
	Lower lobe	67		0.4		0.64				
	Main bronchus/Overlapping lesion/Unknown	5		0.5		0.83				
Tumor size	≤2 cm	122	Reference	<0.01	Reference	<0.01	Reference	0.01	Reference	<0.01
	>2 cm	106	1.59 (1.18–2.16)		1.67 (1.19–2.35)		1.49 (1.10–2.03)		1.65 (1.17–2.33)	
Lymph node involvement	No	213	Reference	<0.01	Reference	0.02	Reference	<0.01	Reference	<0.01
	Yes	15	2.81 (1.58–5.01)		2.35 (1.18–4.67)		2.93 (1.64–5.24)		2.50 (1.25–5.01)	
Histology	Adenocarcinoma	75	Reference	0.05		0.09		0.13		0.22
	Squamous cell carcinoma	106	1.13 (0.79–1.61)	0.51		0.47		0.61		0.60
	Others	47	1.66 (1.10–2.52)	0.02		0.03		0.05		0.09
Differentiation grade	Well/moderately differentiated	81	Reference	0.03	Reference	0.01		0.88		0.33
	Poorly differentiated/ undifferentiated	70	1.22 (0.84–1.78)	0.31	1.63 (1.06–2.50)	0.03		0.66		0.23
	Unknown	77	1.63 (1.13–2.35)	0.01	1.83 (1.19–2.81)	0.01		0.98		0.94
SPLC surgery	No	123	Reference	<0.01	Reference	<0.01	Reference	<0.01	Reference	<0.01
	Yes	105	0.44 (0.32–0.61)		0.54 (0.38–0.77)		0.42 (0.31–0.59)		0.53 (0.37–0.75)	
SCLC^b^ stage	Limited stage	186		0.33		0.86				
	Extensive stage	42								
SCLC surgery	No	194	Reference	0.03		0.06	Reference	0.05		0.10
	Yes	34	0.58 (0.36–0.93)				0.61 (0.38–0.99)			
SCLC chemotherapy	No	28		0.06		0.13				
	Yes	200								
SCLC radiotherapy	No	59		0.09	Reference	0.03				
	Yes	169			1.59 (1.05–2.42)					

a Variables of non-small cell lung cancer if not otherwise specified;

b *SCLC, small cell lung cancer*.

**Figure 2:**
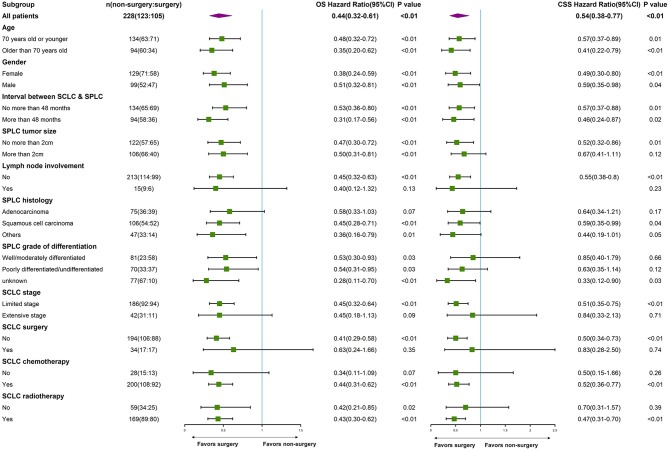
Subgroup analysis by age, gender, interval between cancers, SPLC tumor size, SPLC differentiation grade, SCLC stage, SCLC surgery, SCLC chemotherapy, and SCLC radiotherapy. (a) SPLC: second primary non-small cell lung cancer after SCLC. (b) SCLC: small cell lung cancer.

Subgroup analysis by age, gender, interval between cancers, SPLC tumor size, SPLC differentiation grade, SCLC stage, SCLC surgery, SCLC chemotherapy, and SCLC radiotherapy demonstrated that surgery for SPLC was associated with improved survivals for most subgroups (forest plot, Figure 2).

Among the 105 patients in the surgery group, 43.8% underwent sublobar resection and 51.4% underwent lobectomy, and no statistically significant survival difference was found between sublobar resection and lobectomy (OS, 5-year survival: 41.0 vs. 45.3%, *P* = 0.73; CSS, 5-year survival: 43.5 vs. 54.1%, *P* = 0.49). Regarding the lymph node evaluation during surgery for SPLC, the median number of evaluated lymph node during surgery was 2 and 69.5% patients had at least one lymph node examined during surgery. However, there is no statistically significant survival difference between patients with and without surgical lymph node evaluation (OS, 5-year survival: 30.3 vs. 45.8%, *P* = 0.38; CSS: 5-year survival: 33.1 vs. 52.6%, *P* = 0.29).

Predictor covariates used in PSM included age, gender, marital status, tumor size, lymph node involvement, surgery for SCLC, and interval between cancers. It should be noted that SPLC chemotherapy or radiotherapy was not chosen as predictor covariate in PSM, because they had strong correlation with SPLC surgery and might cause multicollinearity problem. After 1:1 PSM, 162 patients were selected for further analysis (selection flow, Figure 1). No statistically significant difference was found between two groups in predictor covariates or other clinically significant covariates after PSM, except for SPLC differentiation grade (Table 3). It should be noted that differentiation grade was considered a pathological variable and was best acquired from resected sample from surgery, which made the percentage of unknown differentiation grade significantly higher in the non-surgery group. We believed that the difference of differentiation grade of the two groups was mainly derived from surgery. Therefore, differentiation grade was not included as predictor covariates in PSM and did not compromise the comparison between the non-surgery group and the surgery group. Moreover, in the multivariate Cox regression, differentiation grade was not associated with any statistically significant survival difference.

**Table 3:** Comparison of clinicopathological factors between the non-surgery group and surgery group after propensity score matching.

		**Non-surgery group**		**Surgery group**		***P***
		***n***	**Percentage**	***n***	**Percentage**	
Age	≤70 years old	49	60.5%	52	64.2%	0.63
	>70 years old	32	39.5%	29	35.8%	
Gender	Female	45	55.6%	46	56.8%	0.87
	Male	36	44.4%	35	43.2%	
Ethnicity^@^^,^^*^	Caucasian	70	86.4%	73	90.1%	0.5
	African American	9	11.1%	5	6.2%	
	Others	2	2.5%	3	3.7%	
Marital status at SPLC^a^	Married	39	48.1%	42	51.9%	0.64
	Not married	42	51.9%	39	48.1%	
Interval between IPLC and SPLC	≤48 months	54	66.7%	52	64.2%	0.74
	>48 months	27	33.3%	29	35.8%	
SPLC laterality	Left	38	46.9%	29	35.8%	0.15
	Right	43	53.1%	52	64.2%	
Laterality relationship between SPLC and SCLC^@^^,^^*^^,^^b^	Same	31	38.3%	36	44.4%	0.33
	Different	46	56.8%	44	54.3%	
	NA	4	4.9%	1	1.2%	
SPLC primary site^@^^,^^*^	Upper lobe	58	71.6%	48	59.3%	0.09
	Middle lobe	0	0.0%	5	6.2%	
	Lower lobe	22	27.2%	27	33.3%	
	Main bronchus/Overlapping lesion/Unknown	1	1.2%	1	1.2%	
SCLC primary site^@^^,^^*^	Upper lobe	44	54.3%	48	59.3%	0.80
	Middle lobe	2	2.5%	2	2.5%	
	Lower lobe	17	21.0%	18	22.2%	
	Main bronchus/Overlapping lesion/Unknown	18	22.2%	13	16.0%	
SPLC tumor size	≤2 cm	49	60.5%	43	53.1%	0.34
	>2 cm	32	39.5%	38	46.9%	
Lymph node involvement	No	75	92.6%	76	93.8%	0.76
	Yes	6	7.4%	5	6.2%	
SPLC stage	IA–IB	68	84.0%	72	88.9%	0.36
	IIA–IIIA	13	16.0%	9	11.1%	
SPLC histology^*^	Adenocarcinoma	29	35.8%	26	32.1%	0.51
	Squamous cell carcinoma	36	44.4%	43	53.1%	
	Others	16	19.8%	12	14.8%	
SPLC differentiation grade^*^	Well/moderately differentiated	20	24.7%	42	51.9%	<0.01
	Poorly differentiated/undifferentiated	23	28.4%	30	37.0%	
	unknown	38	46.9%	9	11.1%	
SCLC stage	Limited stage	67	82.7%	64	79.0%	0.55
	Extensive stage	14	17.3%	17	21.0%	
SCLC surgery	No	74	91.4%	69	85.2%	0.22
	Yes	7	8.6%	12	14.8%	
SCLC chemotherapy	No	9	11.1%	11	13.6%	0.63
	Yes	72	88.9%	70	86.4%	
SCLC radiotherapy	No	20	24.7%	22	27.2%	0.72
	Yes	61	75.3%	59	72.8%	

a SPLC, second primary non-small cell lung cancer after small cell lung cancer;

b SCLC, small cell lung cancer;

@ At least one of the cells had expected cell count <5, Fisher's exact test was used;

* *Multiple comparisons were adjusted by Bonferroni correction*.

**Figure 3:**
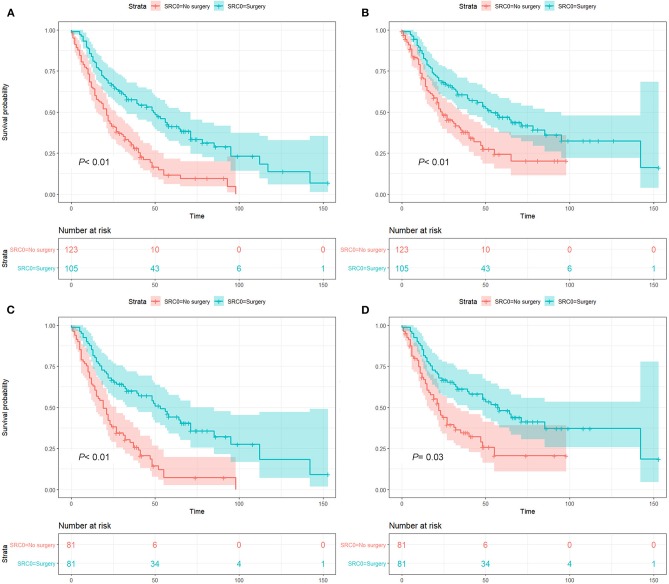
Survival analysis before and after propensity score matching (PSM): before PSM, overall survival **(A)** and lung cancer-specific survival **(B)** analysis; after PSM, overall survival **(C)** and lung cancer-specific survival **(D)** analysis. *P* value was calculated from log-rank test and pooled over strata, and the 95% confidence interval of the survival curves is depicted as a color band.

Notably, patients who underwent surgery demonstrated significantly improved survival both before (OS, 5-year rate: 41.2 vs. 11.6%, HR: 0.44, 95% CI: 0.32–0.61, *P* < 0.01; CSS, 5-year rate: 46.8 vs. 24.3%, HR: 0.54, 95% CI: 0.38–0.77, *P* < 0.01, Figures 3A,B) and after PSM (OS, 5-year rate: 44.2 vs. 7.2%, HR: 0.48, 95% CI: 0.33–0.70, *P* < 0.01; CSS, 5-year rate: 48.0 vs. 20.6%, HR: 0.44, 95% CI: 0.42–0.97, *P* = 0.03, Figures 3C,D) compared to patients without surgery for SPLC.

## Discussion

Although the survival rate of SCLC remains dismal, certain number of patients survived long enough to develop second primary malignancies, including NSCLC (4, 5). However, the optimal management strategy for these patients remains unknown, and to the best of our knowledge, no previous study has evaluated the survival benefit of surgery in these patients. In this study, we utilized the SEER database to identify the largest cohort of early-stage SPLC after SCLC, and we found that surgery was associated with significantly better OS and CSS in these patients. To further confirm the survival benefits of surgery, we also conducted multivariate Cox regression, subgroup analysis, and PSM. As it turned out, surgery demonstrated consistent survival benefits in the multivariate Cox regression and most subgroups, indicating a wide range of application in early-stage SPLC after SCLC. Furthermore, surgery was also associated with better OS and CSS after PSM.

Not so many thoracic surgeons recommend surgery for early-stage SPLC after SCLC, especially after chemoradiation for the initial SCLC, as the dissection of pulmonary vessels can be challenging and technically demanding after definitive chemoradiation. Although studies on surgery after SCLC chemoradiation have been scarce, multiple studies have demonstrated that salvage surgery after failed Stereotactic Body Radiotherapy in early-stage NSCLC was safe and feasible and led to improved survival (10–12). A phase III randomized controlled trial also found that resection after induction chemoradiation was feasible and comparable to definitive chemoradiation in patients with locally advanced NSCLC (13). In our study, most patients received chemotherapy and radiotherapy for the initial SCLC. Similar to the studies described above, we believe that surgery for SPLC after SCLC can be safe, feasible, and beneficial in experienced hands.

The extent of resection and lymph node evaluation during surgery are two important aspects of NSCLC surgery. Previous studies have provided convincing evidence that recommended lobectomy over sublobar resection as standard of care for early-stage NSCLC (14–16), based on the associated longer survival and lower recurrence. On the other hand, the number of examined lymph node was also found to be an important prognostic factor (17, 18), and thorough lymph node evaluation may avoid downstaging. However, in our study, neither the extent of resection nor the number of examined lymph node was a prognostic factor within the surgery group. We believe that the decision on the extent of resection and lymph node evaluation should be individually based for patients with early-stage SPLC after SCLC. Multiple factors, including patients' remaining pulmonary function, life expectancy, operative risk, and surgeon's experience should be considered. Moreover, further randomized controlled trials are needed to determine the optimal extent of resection and lymph node evaluation in patients with early-stage SPLC after SCLC.

Several limitations in our study are worth mentioning. First, several important variables are not available in SEER, including cardiopulmonary function, specific protocols of chemotherapy and radiotherapy, SCLC treatment response, and perioperative morbidity and mortality. Second, potential bias is inevitable due to the retrospective nature of this study. However, it should be noted that multivariate Cox regression and PSM were implemented to minimize this potential bias.

In conclusion, this population-based study has demonstrated that surgery is associated with significantly improved survival. Therefore, surgery should be considered in patients with early-stage SPLC after SCLC whenever possible. Nevertheless, further randomized controlled trials are warranted to confirm the safety and effect of surgery in these patients.

## Data Availability Statement

All data in this study are publicly available in the SEER database: Multiple Primary Standardized Incidence Ratios (MP-SIR) session (April 2019 released, 2000–2016).

## Author Contributions

RZ, LC, YL, and LZ: conception and design. YW, NZ, FW, XZ, YL, and LZ: provision of study materials or patients. GW, ZH, XY, KX, LY, and DZ: collection and assembly of data. RZ and LC: data analysis and interpretation. All authors: manuscript writing and final approval of manuscript.

### Conflict of Interest

The authors declare that the research was conducted in the absence of any commercial or financial relationships that could be construed as a potential conflict of interest.
